# Intranasal Chitosan Nano-Formulations: A Novel Frontier in Brain Cancer Therapy

**DOI:** 10.3390/polym18212723

**Published:** 2026-11-02

**Authors:** Sina Matalqah, Zainab Lafi, Randa Atwan, Sara Yousef Asha, Asem N. Mhaidat

**Affiliations:** 1 Pharmacological and Diagnostic Research Center, Faculty of Pharmacy, Al-Ahliyya Amman University, Amman 19328, Jordan; ratwan@ammanu.edu.jo; 2 Department of Pharmaceutics and Pharmaceutical Technology, Faculty of Pharmacy, Al-Ahliyya Amman University, Amman 19328, Jordan; 3 School of Medicine, University of Jordan, Amman 11942, Jordan; sarah.asha20@gmail.com; 4 King Abdullah University Hospital, Irbid 22110, Jordan; asem412003@gmail.com

**Keywords:** chitosan, intranasal drug delivery, nose-to-brain delivery, nanocarriers, glioblastoma multiforme, brain tumors, blood–brain barrier, mucoadhesion, temozolomide, RNA therapeutics

## Abstract

Direct nose-to-brain drug delivery is emerging as a promising approach in neuro-oncology because it offers a non-invasive, needle-free route for delivering therapeutics to the brain while partially bypassing the blood–brain barrier (BBB). This approach takes advantage of the natural connections between the nasal cavity and the central nervous system, allowing drugs administered through the nose to reach brain tissues through the olfactory and trigeminal pathways. Despite its potential, successful clinical translation remains challenging because the nasal cavity presents several physiological barriers. Mucus can trap drug molecules, mucociliary clearance can rapidly remove them, and nasal enzymes can degrade them before they reach their target. Advanced nanocarrier systems are therefore being developed to overcome these barriers and improve drug transport through intracellular, paracellular, and transcellular pathways. In this context, specific structural modifications can enhance drug retention, mucus penetration, cellular uptake, and targeting efficiency. Important strategies include the use of mucoadhesive materials such as chitosan to increase residence time in the nasal cavity; hydrophilic coatings such as polyethylene glycol (PEG) to improve movement through mucus; targeting ligands to promote selective cellular uptake; and stimuli-responsive systems that respond to local conditions such as pH, redox state, or glutathione (GSH) levels. These advances are particularly relevant to the treatment of glioblastoma multiforme (GBM), one of the most aggressive and difficult-to-treat brain tumors. GBM is characterized by extensive infiltration into surrounding healthy brain tissue, making it difficult to achieve effective drug concentrations throughout both the main tumor mass and its invasive margins. Intranasally delivered chitosan-based nano-formulations may help address this problem through a series of transport steps. After administration, the nanoparticles can interact with and remain within the nasal mucosa, move across the olfactory epithelium, and pass through the cribriform plate toward the olfactory bulb. From there, they may distribute into deeper regions of the brain and potentially increase drug accumulation within both the GBM core and infiltrative tumor regions. This review presents a structured overview of engineered chitosan nano-formulations for targeted drug delivery in neuro-oncology. It focuses on their physicochemical properties, mechanisms of nose-to-brain transport, drug-loading strategies, tumor-targeting approaches, therapeutic applications, and the major challenges that must be addressed before clinical translation. Overall, these systems offer a promising strategy for improving the precision and effectiveness of drug delivery to brain tumors and may contribute to the development of more targeted treatments for GBM and other neurological malignancies.

## 1. Introduction

Glioblastoma (GBM), the most aggressive and prevalent malignant primary brain tumor in adults, remains a major challenge in neuro-oncology because of its highly infiltrative growth, molecular heterogeneity, treatment resistance, and propensity for recurrence. Standing as the most frequent adult central nervous system primary malignancy, GBM accounts for approximately 48% of all malignant brain tumor cases. On a global scale, primary brain and CNS malignancies are responsible for over 324,000 new cases and approximately 248,000 annual deaths, carrying a devastating 5-year mortality rate that exceeds 90% [1,2]. Despite aggressive interventions consisting of maximally safe surgical resection followed by concurrent radiotherapy and temozolomide (the standard-of-care Stupp protocol), the median overall survival remains constrained to a dismal 14 to 18 months, with local tumor recurrence being virtually inevitable [2]. The addition of temozolomide to radiotherapy has improved survival compared with radiotherapy alone, but the overall benefit remains limited, particularly for patients with recurrent or treatment-resistant disease [3,4]. This poor prognosis is profoundly driven by the blood–brain barrier (BBB) and the blood–brain–tumor barrier (BBTB), which restrict more than 98% of small-molecule chemotherapeutics and nearly 100% of large-molecule targeted agents from accumulating within the infiltrative tumor margins. Consequently, developing a non-invasive administration route that can bypass systemic restrictions entirely is of paramount clinical importance [1,5]. This limitation has encouraged the development of alternative drug-delivery strategies that can bypass or overcome the BBB.

Among these approaches, intranasal, or nose-to-brain, drug delivery has gained considerable interest as a non-invasive method for transporting therapeutics directly from the nasal cavity to the central nervous system. This route can utilize the olfactory and trigeminal neural pathways, reducing reliance on systemic circulation and potentially improving drug accumulation within the brain [6,7]. However, the effectiveness of conventional intranasal formulations is often limited by rapid mucociliary clearance and their short residence time in the nasal cavity. These challenges highlight the need for specially designed nanocarriers that can improve nasal retention, enhance drug transport, and protect therapeutic agents from premature removal or degradation.

In this context, chitosan has attracted considerable attention as a promising material for intranasal brain drug delivery. Chitosan is a naturally derived, biodegradable, biocompatible, and positively charged polysaccharide with several properties that make it suitable for nanocarrier development. Its mucoadhesive and permeability-enhancing properties can help prolong drug residence in the nasal cavity and facilitate transport across the nasal mucosa, making chitosan-based nanocarriers an attractive platform for nose-to-brain delivery and targeted treatment of brain tumor. Its positive surface charge promotes interaction with negatively charged mucin and nasal epithelial surfaces, thereby enhancing mucoadhesion and prolonging residence within the nasal cavity, while its ability to transiently modulate epithelial tight junctions may facilitate drug permeation [8,9,10]. Moreover, chitosan nanoparticles can provide drug encapsulation, protection against degradation, controlled release, and opportunities for surface functionalization with targeting ligands, thereby supporting the development of high-payload and tumor-directed therapeutic systems [8,11]. Recent evidence further demonstrates that engineered chitosan nanoparticles can enhance nose-to-brain transport and brain targeting, while functionalization with ligands such as transferrin may provide an additional level of cellular and molecular targeting [12,13]. Therefore, intranasal chitosan-based nanocarriers represent a promising platform for overcoming key physiological and pharmacological barriers in brain cancer therapy. This review provides a structured overview of the design and therapeutic potential of engineered chitosan nano-formulations for targeted neuro-oncology, with particular emphasis on their physicochemical characteristics, mechanisms of nose-to-brain transport, drug-loading strategies, tumor-targeting approaches, therapeutic applications, and translational challenges, with the ultimate goal of defining how these systems may contribute to more effective and precise treatment of brain malignancies.

While the direct nose-to-brain (N2B) route via the olfactory and trigeminal nerve pathways has long been proposed as an alternative vector, the past 2–3 years have witnessed an exceptional shift in the material design of mucosal carriers. Historically, standard chitosan polymer matrices struggled with rapid mucociliary clearance and poor specificity within the complex tumor architecture. However, recent breakthroughs have successfully transitioned the field into an era of surface-engineered, stimuli-responsive, and multi-functionalized chitosan nano-formulations. Emerging strategies now employ sophisticated ligand-conjugation (such as chlorotoxin or transferrin) and transient tight-junction modulations to actively home into the glioblastoma microenvironment while drastically extending nasal residence times. Moreover, the therapeutic payloads encapsulated within these platforms have evolved from simple cytotoxins to complex genetic materials (siRNA, miRNA) and personalized immunotherapies [6,7].

### Scope and Distinctive Novelty of This Review

The rapid evolution of biomaterial science in neuro-oncology has led to several excellent recent syntheses of literature, which this review builds upon rather than duplicates.

Specifically, Khodaverdi et al. (2024) provided a broad overview of chitosan nanocarriers across a wide spectrum of central nervous system disorders [5]. Riani et al. (2026) thoroughly reviewed general nose-to-brain mechanics utilizing chitosan platforms [6]. Sarkar et al. (2025) detailed the systemic administration routes of chitosan nanocarriers specifically for glioblastoma therapy [7]. Leslie et al. (2026) addressed the critical clinical translation and regulatory bottlenecks facing intranasal nanomedicines [8].

In contrast to these existing works, the present review occupies a unique niche at the exact intersection of these fields. It provides the first dedicated, systematic evaluation focusing exclusively on how surface-engineered chitosan nanocarriers can be customized to simultaneously overcome mucosal intranasal clearance and bypass the highly restrictive glioblastoma microenvironment via the direct nose-to-brain axis.

## 2. Materials and Methods

### 2.1. Literature Search Strategy

A literature search was conducted to identify studies investigating chitosan-based intranasal nanocarriers for brain drug delivery and their potential applications in brain cancer. Relevant literature was searched in PubMed, Scopus, and Web of Science, with Google Scholar used as a supplementary search source. The search covered publications from database inception to the search covered publications to September 2026. Search terms included combinations of the following keywords: “chitosan,” “intranasal delivery,” “nose-to-brain delivery,” “nanocarriers,” “nanoparticles,” “glioblastoma,” “brain tumors,” “blood–brain barrier,” “temozolomide,” and “RNA-based therapeutics.” Boolean operators (AND, OR) were used to combine search terms and broaden or refine the results.

Research articles and relevant review articles were considered, while unrelated publications, duplicate records, and studies without sufficient relevance to the review objectives were excluded. Reference lists of selected publications were also examined to identify additional relevant studies. The retrieved literature was assessed for its relevance to formulation characteristics, drug delivery mechanisms, therapeutic applications, safety, and translational challenges. This search was designed to support a narrative review rather than a systematic review or meta-analysis.

### 2.2. Bibliometric and Keyword Co-Occurrence Analysis

To identify major research themes and emerging trends in the field, a bibliometric analysis was performed using VOSviewer software (version 1.6.21). Bibliographic records were exported from the selected databases and prepared for analysis by removing duplicate records and standardizing keyword terminology where appropriate. Keyword co-occurrence analysis was conducted to identify frequently occurring terms and their relationships within the literature. The analysis focused on author keywords and, where supported by the available records, keywords assigned by database indexers.

**Figure 1:**
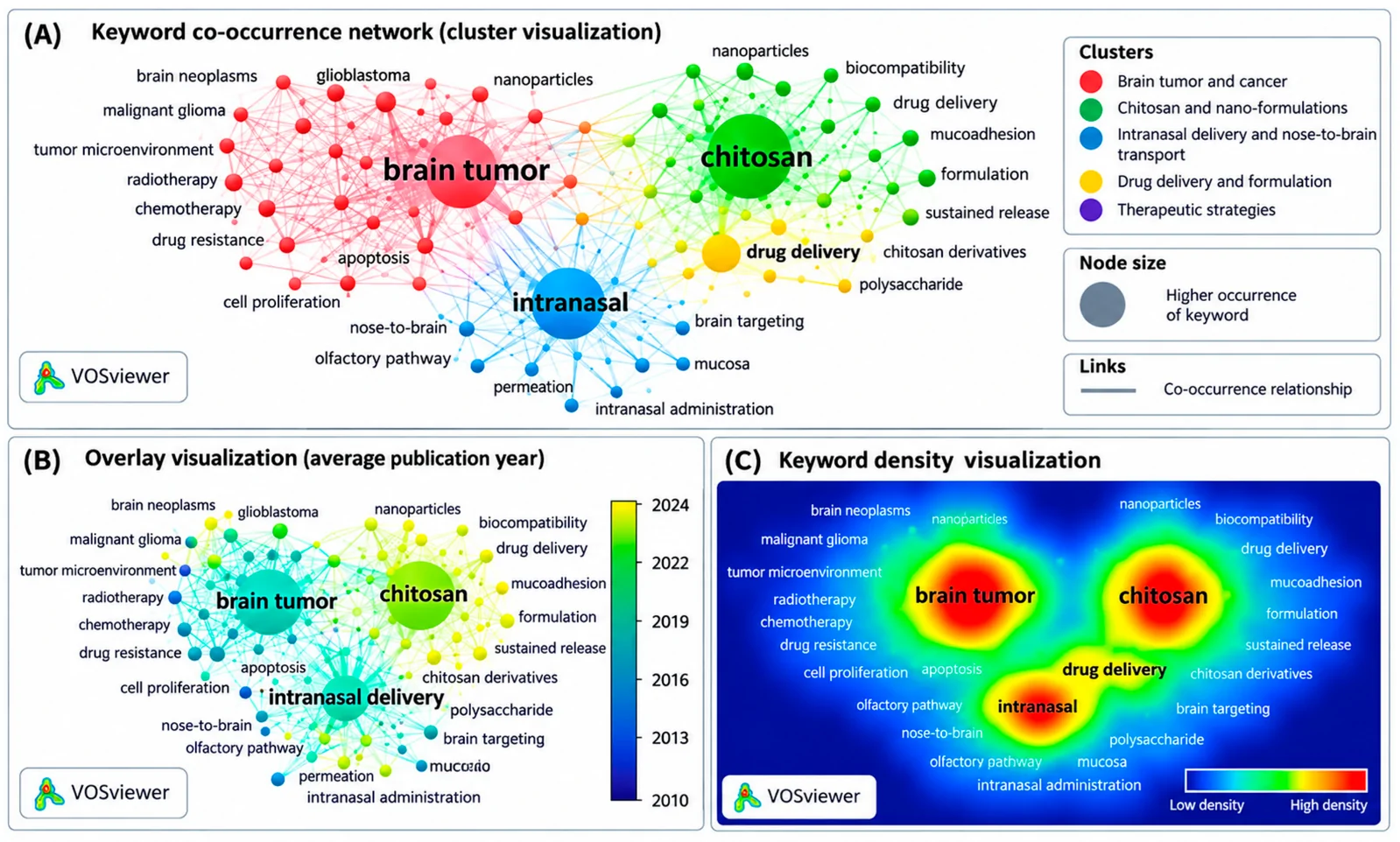
Bibliometric analysis of keyword networks related to intranasal chitosan nano-formulations for brain cancer therapy. (**A**) Keyword co-occurrence network (cluster visualization) demonstrating the major thematic clusters, where node size corresponds to the occurrence frequency of the keyword and line thickness indicates the strength of the co-occurrence relationship. (**B**) Overlay visualization mapping the evolution of the research landscape based on the average publication year, highlighting a recent shift toward advanced targeted nano-formulations. (**C**) Keyword density visualization highlights the most densely saturated and highly interconnected core topics in current literature, with hot zones indicating primary research focus areas. Maps generated and visualized using VOSviewer software.

A keyword co-occurrence network was constructed to identify clusters representing related research themes. The minimum keyword occurrence threshold was set at [insert threshold], and the selection of terms was based on their frequency and relevance to the review topic. VOSviewer was used to visualize the network structure, with nodes representing keywords and links indicating their co-occurrence relationships. Overlay visualization was also used to examine the average publication year associated with individual keywords and to identify changes in research emphasis over time. These analyses provided a complementary overview of established research areas, emerging topics, and potential directions for future research in chitosan-based intranasal nanocarriers for GBM treatment (Figure 1).

## 3. Anatomy and Physiology of Nose-to-Brain Pathways in Oncology

The anatomical connection between the nasal cavity and the central nervous system (CNS) provides a distinctive opportunity for non-invasive delivery of therapeutics to the brain while partially circumventing the systemic circulation and blood–brain barrier (BBB). Following intranasal administration, drug-loaded nanoparticles can access the CNS through direct neuronal pathways, principally the olfactory and trigeminal routes, as well as through indirect vascular and lymphatic mechanisms [14,15,16]. The olfactory pathway originates in the specialized olfactory epithelium located in the superior nasal cavity, where the axons of olfactory sensory neurons traverse the cribriform plate and terminate in the olfactory bulb. Therapeutic molecules or nanoparticles deposited in this region may reach the brain through extracellular transport along olfactory nerve bundles, transcellular uptake by olfactory neurons, or, to a lesser extent, intracellular axonal transport [15,17]. The trigeminal pathway provides an additional anatomical connection because branches of the ophthalmic and maxillary divisions of the trigeminal nerve innervate both the olfactory and respiratory regions of the nasal mucosa and project toward the brainstem and more extensive CNS regions [15,18]. These neuronal routes are particularly relevant to neuro-oncology because they provide a potential mechanism for delivering anticancer therapeutics without requiring extensive passage through the BBB. In parallel, indirect pathways may involve absorption across the nasal epithelium into the local vasculature, followed by systemic circulation and subsequent BBB transport, whereas lymphatic drainage and cerebrospinal fluid (CSF)-associated pathways may also contribute to CNS exposure [16,19].

The relative contribution of these routes remains formulation- and species-dependent and should therefore be distinguished from true direct nose-to-brain transport.

The efficiency of nose-to-brain delivery is nevertheless constrained by several anatomical and physiological barriers within the nasal cavity. The nasal mucosa is covered by a continuously renewed mucus layer that traps foreign materials and facilitates their removal toward the nasopharynx through coordinated ciliary movement, a process known as mucociliary clearance [20]. Consequently, conventional liquid formulations may have a short residence time at the olfactory region, reducing the fraction of drug available for CNS transport. In addition, enzymatic activity within the nasal mucosa can contribute to degradation of susceptible drugs and biological macromolecules before they reach the underlying epithelium [20,21,22]. However, enhancement of epithelial permeability must be carefully controlled because excessive disruption of epithelial tight junctions may compromise mucosal integrity and increase local toxicity.

From an oncological perspective, the most important question is whether successful nasal deposition translates into meaningful distribution within the tumor and its infiltrative margins. GBM presents a particularly difficult target because malignant cells extensively infiltrate surrounding normal brain tissue and may be located far beyond the central tumor mass. Thus, delivery to the olfactory bulb or superficial anterior brain does not necessarily imply adequate penetration into deeper tumor regions. Experimental evidence nevertheless demonstrates that appropriately engineered intranasal nanocarriers can achieve measurable brain and tumor exposure. For example, Ahmad et al. developed chitosan-coated PLGA nanoparticles containing carmustine and demonstrated that intranasal administration produced substantially greater brain exposure than both intranasal carmustine suspension and intravenous administration, with brain AUC values approximately 2.8-fold and 14.7-fold higher, respectively [23]. Particularly relevant to targeted neuro-oncology, Van et al. reported that intranasally administered siRNA-loaded chitosan nanoparticles targeting Galectin-1 reached the CNS tumor within hours and enabled delivery of siRNA to GBM tissue, demonstrating the potential of chitosan nanoparticles to transport molecular therapeutics toward intracranial tumors [24]. These findings suggest that nanoparticle deposition at the olfactory and trigeminal interfaces may initiate transport toward anatomically distinct brain regions; however, the extent to which nanoparticles penetrate deep tumor structures, infiltrative GBM margins, and heterogeneous tumor microenvironments remains an important area requiring systematic investigation. Consequently, future studies should combine whole-brain biodistribution imaging, quantitative tumor-to-normal-brain ratios, spatial mapping of nanoparticles within tumor margins, and pharmacodynamic assessment to establish whether nose-to-brain delivery produces therapeutically meaningful concentrations throughout the GBM microenvironment.

## 4. Mechanisms of Chitosan-Mediated Anti-Tumor Delivery

### 4.1. Electrostatic Mucoadhesion

**Figure 2:**
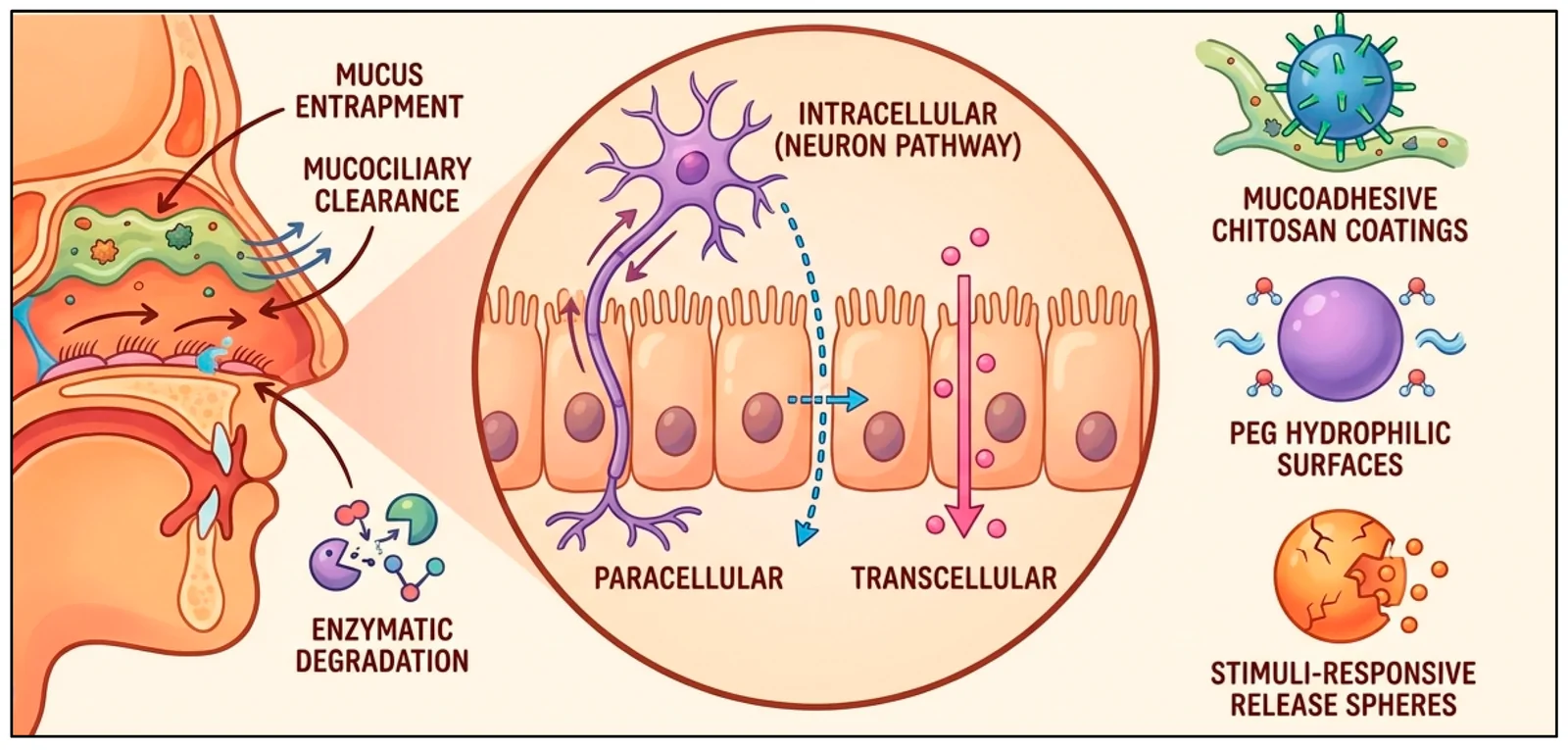
Schematic illustration of the mechanisms and barriers involved in intranasal nanocarrier delivery for brain-targeted drug delivery. The illustration shows the major challenges encountered following intranasal administration, including mucus entrapment, mucociliary clearance, and enzymatic degradation. It also depicts the possible transport pathways across the nasal epithelium, including paracellular and transcellular transport, as well as intracellular neuronal transport. In addition, different nanocarrier surface modifications and formulation strategies are illustrated, including mucoadhesive chitosan coatings, polyethylene glycol (PEG)-modified hydrophilic surfaces, and stimuli-responsive nanocarriers, which may improve drug delivery to the brain.

The strong mucoadhesive properties of chitosan are primarily associated with its cationic nature under mildly acidic conditions. Protonation of the primary amino groups of chitosan, which possess a pKa of approximately 6.5, generates positively charged, NH_3_^+^ groups that can interact electrostatically with negatively charged components of mucin, particularly sialic acid and sulfate-containing residues. These interactions, together with hydrogen bonding and hydrophobic interactions, promote intimate association between chitosan-based nanocarriers and the nasal mucus layer, thereby increasing their residence time and reducing rapid mucociliary clearance [8] (Figure 2).

However, the extent of mucoadhesion heavily depends on critical factors such as polymer concentration, pH, ionic strength, degree of deacetylation (DD), and chitosan molecular weight (MW). Specifically, a high degree of deacetylation—typically exceeding 75% to 85%—is essential to maximize cationic charge density, driving up to a several-fold increase in the initial electrostatic binding force with the mucosal lining. Concurrently, the molecular weight of the polymer modulates the secondary phase of physical chain entanglement. While low molecular weight chitosan (<150 kDa) provides rapid diffusion and swelling, it lacks the chain length required for durable binding [25]. Elevating the molecular weight to medium (150–700 kDa) or high (>700 kDa) ranges provides the necessary chain interpenetration and configuration to maximize mechanical detachment forces, successfully extending nasal residence times from a baseline of 15 min to several hours. This prolonged nasal residence is particularly relevant to intranasal nose-to-brain delivery, because it provides more time for nanoparticles to interact with the olfactory and trigeminal regions and facilitates subsequent transport toward the central nervous system [26].

### 4.2. Paracellular Modulation

In addition to mucoadhesion, chitosan can enhance epithelial permeability through transient modulation of intercellular tight junctions. The positively charged polymer interacts with negatively charged components of epithelial cell membranes and can induce structural redistribution of tight-junction proteins, thereby increasing the paracellular transport of therapeutic molecules and nanoparticles [27]. Experimental studies have demonstrated that chitosan can produce a reversible reduction in transepithelial electrical resistance accompanied by transient tight-junction opening; mechanistic investigations have implicated redistribution and lysosomal degradation of claudin-4 in this process. In the nasal cavity, this permeation-enhancing effect may facilitate transport across the nasal epithelium and complement the mucoadhesive properties of chitosan. Importantly, the magnitude and reversibility of tight-junction modulation depend on the physicochemical characteristics and concentration of the chitosan system, and excessive epithelial disruption should be avoided to maintain mucosal integrity [25].

### 4.3. Ph-Responsive Payload Release

The protonation behavior of chitosan provides an important basis for developing pH-responsive nanocarriers for cancer therapy. Chitosan has an apparent pKa generally reported around 6.2–6.5; consequently, its amino groups become increasingly protonated as the environmental pH decreases. In appropriately engineered formulations, this protonation can increase electrostatic repulsion between polymer chains, induce swelling or partial dissolution of the chitosan matrix, weaken interactions between chitosan and the encapsulated drug or cross-linking agent, and consequently accelerate payload diffusion [28]. This property can be exploited in tumor-responsive systems because the extracellular tumor microenvironment is generally more acidic than normal physiological tissue, while substantially lower pH values occur within endosomal and lysosomal compartments after cellular internalization. Importantly, acidic pH does not automatically result in drug release from every chitosan nanoparticle; the response depends on the cross-linking chemistry, drug–polymer interactions, particle architecture, and any additional pH-sensitive components incorporated into the formulation. Experimental studies have demonstrated enhanced release of anticancer agents from engineered chitosan systems under acidic conditions, supporting the development of pH-responsive platforms for intracellular and tumor-associated drug release [29].

### 4.4. Enhanced Intracellular Uptake MDR

Chitosan nanoparticles can facilitate intracellular drug delivery through electrostatic interactions with the negatively charged cellular membrane, followed by endocytic internalization. Their nanoscale dimensions and modifiable surface chemistry allow drugs, nucleic acids, and other therapeutic cargos to enter cells through pathways that differ from the passive diffusion mechanisms of many free drugs [30]. Once internalized, appropriately designed chitosan nanocarriers may increase intracellular drug accumulation and, in some systems, reduce exposure of the free drug to membrane-associated efflux transporters such as P-glycoprotein (P-gp). For example, N-octyl-O-sulfate chitosan micelles containing paclitaxel demonstrated increased cellular uptake and reduced drug efflux in P-gp-overexpressing cells, whereas glycol chitosan nanoparticles carrying MDR-1 siRNA were shown to downregulate P-gp expression and improve the response to chemotherapy in an experimental drug-resistant breast cancer model [31]. Thus, chitosan nanocarriers may contribute to the overcoming of MDR through several mechanisms, including enhanced endocytic uptake, intracellular drug release, transporter modulation, and delivery of nucleic acids that suppress resistance-associated genes. Nevertheless, chitosan nanoparticles should not be considered universally capable of bypassing MDR pumps; the effect is highly dependent on nanoparticle composition, surface modification, cargo, cellular uptake pathway, and the specific resistance mechanism involved [32].

## 5. Surface Modifications and Engineering of Chitosan Nanoparticles

### 5.1. Thiolated Chitosan and Trimethyl Chitosan: Advanced Chemical Adaptations

Chemical modification of chitosan provides an effective strategy for overcoming limitations associated with its native physicochemical properties and for tailoring nanoparticle performance for specific administration routes [33,34]. Thiolated chitosan (thiomer) is generated by introducing sulfhydryl-containing groups onto the primary amino backbone of the polymer using specialized reagents such as thioglycolic acid (TGA), 2-iminothiolane (Traut’s reagent), L-cysteine, or N-acetylcysteine. This modification alters the primary binding mechanism from weak physical forces to stable, covalent inter-macromolecular disulfide bonds with the cysteine-rich sub-domains of mucin glycoproteins [35,36].

The mucoadhesive performance of these thiomers is strictly dictated by their degree of chemical modification. Critical evaluation of recent literature establishes that the optimal degree of thiolated modification falls within a narrow window of 150 to 450 μmol of sulfhydryl groups per gram of polymer matrix. Modifications falling below 150 μmol/g yield insufficient cross-linking networks to outperform native chitosan, whereas exceeding 450 μmol/g triggers premature intra- and intermolecular oxidation during formulation, which restricts swelling behavior and hinders mucosal accessibility [37]. In vivo mucoadhesion data underscores the translational efficiency of these optimized platforms; gamma scintigraphy and fluorescent tracking in rodent and porcine models demonstrate that thiolated chitosan nanoparticles successfully extend local nasal residence times from a baseline of 15–20 min to over 4 to 6 h. This sustained localized retention reduces rapid mucociliary clearance, driving a 3- to 5-fold amplification in the absolute central nervous system bioavailability of encapsulated anti-glioblastoma agents across the transmucosal epithelium [36,37].

In contrast, trimethyl chitosan (TMC) represents a structural adaptation targeting solubility limitations rather than covalent bonds. TMC is a permanently cationic, quaternized derivative that retains its positive charge density over a broader physiological pH range than native chitosan, independent of the local environment. Because native chitosan loses its charge as pH approaches its (Pka ~6.5), TMC’s permanent quaternization consequently exhibits drastically improved aqueous solubility under neutral and alkaline physiological conditions while maintaining powerful electrostatic mucoadhesive and tight-junction permeation-enhancing properties. Taken together, these dual modifications are uniquely relevant for nasal nanocarriers in neuro-oncology, where prolonged mucosal retention, targeted neural uptake, and robust polymer solubility must be achieved simultaneously [37].

### 5.2. Active Tumor Targeting

Surface functionalization of chitosan nanoparticles with specific ligands can introduce an active-targeting component that complements the intrinsic properties of the polymer. Ligands such as transferrin, lactoferrin, and chlorotoxin have been investigated because their receptors or molecular targets are expressed or enriched in brain endothelial cells and/or glioma cells [8,38]. Lactoferrin-functionalized chitosan-containing polysaccharide nanoparticles, for example, demonstrated enhanced uptake by brain capillary endothelial cells and increased brain accumulation compared with non-targeted nanoparticles, with evidence of subsequent glioma-cell targeting [39]. Similarly, chlorotoxin-functionalized magnetic nanoparticles incorporating a chitosan-containing coating enhanced nanoparticle uptake by glioma cells in a xenograft model, although chlorotoxin did not itself increase overall nanoparticle accumulation at the tumor site. These findings emphasize that active targeting should be considered a mechanism for improving cellular recognition and uptake rather than being equated automatically with increased tumor accumulation [40]. The effectiveness of a targeting ligand depends on receptor abundance, ligand density and orientation, nanoparticle size and surface charge, biological barriers, and the heterogeneity of glioblastoma [40].

### 5.3. Biomimetic and Hybrid Platforms

For example, chitosan nanoparticles coated with engineered macrophage membranes expressing transmembrane TNF-α demonstrated anticancer activity against several cancer cell lines and tumor spheroids, illustrating the potential of macrophage-derived membranes to impart biological functionality to chitosan nanocarriers. In parallel, chitosan–lipid hybrid systems combine the mucoadhesive and permeation-enhancing characteristics of chitosan with the membrane-interacting and drug-encapsulation properties of lipid components. Such hybrid architecture can improve nanoparticle stability and drug-delivery performance and can accommodate therapeutic molecules with different physicochemical properties. Chitosan-coated or chitosan-containing lipid nanocarriers have therefore been investigated for controlled, targeted, and sustained drug delivery [41]. Biomimetic and hybrid approaches extend the functionality of chitosan nanoparticles by combining the polymer with biological membranes or complementary lipid/polymeric components. Cell-membrane-coated nanoparticles, including macrophage- and platelet-membrane systems, can exploit naturally occurring membrane proteins to improve biological interactions, prolong circulation, reduce premature immune recognition, or enhance tumor-cell association. For example, platelet-membrane-coated nanoparticles containing a chitosan oligosaccharide–PLGA core demonstrated enhanced cancer-cell uptake and tumor accumulation compared with the corresponding uncoated nanoparticles, with P-selectin/CD44 interactions contributing to tumor-cell recognition. Macrophage-membrane-coated chitosan nanoparticles have likewise been investigated as biomimetic platforms for tumor-directed delivery [42].

Despite the compelling biological performance of these cell-membrane-wrapped and hybrid chitosan systems, their clinical translation is severely bottlenecked by severe manufacturing scale-up hurdles. Conventional benchtop production heavily relies on multi-step differential centrifugation coupled with physical extrusion or sonication, methods that suffer from low isolation yields and profound batch-to-batch inconsistency. Transitioning to high-throughput, current Good Manufacturing Practice (cGMP) industrial pipelines frequently compromises structural integrity, leading to unencapsulated core populations, variable shell thicknesses, or total inversion of the lipid bilayer. Furthermore, mechanical scale-up forces can denature or shear away the critical surface proteins (such as P-selectin or tumor-homing ligands) necessary for glioblastoma cell-specific recognition, resulting in a loss of targeting functionality.

Sterility assurance presents an equally formidable barrier for these biomimetic architectures. Standard terminal sterilization methodologies are entirely incompatible with cell membrane components; thermal autoclaving completely denatures delicate membrane proteins, while gamma irradiation triggers devastating lipid peroxidation and compromises the structural integrity of the underlying chitosan matrix [42,43,44].

### 5.4. Stimuli-Responsive Elements

Incorporation of stimuli-responsive components into chitosan nanoparticles represents an important strategy for achieving spatially and temporally controlled drug release. Endogenous stimuli, including acidic pH, redox conditions, and tumor-associated enzymes, can be exploited to destabilize specific chemical bonds or alter polymer conformation, whereas exogenous stimuli, including magnetic fields, light, ultrasound, and externally applied heat, can provide additional control over nanoparticle activation [45,46].

Magnetic chitosan nanocomposites are particularly attractive for brain and tumor applications because magnetic cores can provide external magnetic guidance and localized hyperthermia under an alternating magnetic field, while the chitosan component can function as drug-carrying, protective, or responsive shell [47]. Recent studies have also demonstrated magnetic/pH-responsive magnetite–chitosan nanoparticles capable of combining magnetic responsiveness with pH-dependent drug release, illustrating the potential of dual-stimuli systems for cancer therapy.

Similarly, enzyme-responsive chitosan nanoparticles can incorporate cleavable or enzymatically degradable components that facilitate drug release in tumor-associated or intracellular environments. For example, CD44-targeted chitosan nanoparticles incorporating an enzymatically responsive design exhibited enhanced antitumor activity and drug-release behavior, demonstrating how enzyme responsiveness can be integrated with active targeting [48].

Combining two or more stimuli—for example, pH plus magnetic or thermal responsiveness—may provide greater control over drug release than a single trigger. However, increasing structural complexity can also introduce challenges related to formulation reproducibility, physicochemical stability, toxicity, manufacturing scalability, and clinical translation [45,46].

## 6. Therapeutic Applications in Neuro-Oncology

### 6.1. Chemotherapy Re-Engineering

Intranasal administration provides an attractive strategy for re-engineering conventional chemotherapy for glioblastoma (GBM) by enabling direct nose-to-brain transport and partially circumventing the blood–brain barrier (BBB). Chitosan is particularly suitable for this purpose because its cationic and mucoadhesive characteristics can increase residence time at the nasal mucosa, improve interaction with the mucus layer, and facilitate epithelial transport [40]. Temozolomide (TMZ), the standard alkylating agent used in GBM, has been investigated extensively in chitosan-containing intranasal systems. Khan et al. developed a TMZ-loaded nano-lipid chitosan hydrogel with a particle size of approximately 134 nm and reported an IC_50_ of 3.34 μg/mL compared with 160 μg/mL for free TMZ, together with increased nasal permeation and no evident structural damage to nasal mucosa in the histopathological evaluation. More recently, Jain et al. developed chitosan-coated pH-sensitive liposomes for intranasal TMZ delivery and reported a greater than 22-fold increase in brain AUC compared with free TMZ in rats, together with enhanced uptake and cytotoxicity in U87MG cells [49,50].

Paclitaxel (PTX) and doxorubicin (DOX) provide additional examples of chemotherapy re-engineering through the intranasal route. RGD-functionalized PTX-loaded nanoparticles delivered intranasally produced a reported 75 ± 12% reduction in tumor burden in an orthotopic GBM model, demonstrating the potential of combining nose-to-brain delivery with tumor-specific targeting. A recent study further investigated PTX-loaded hybrid nanoparticles with chitosan coating and demonstrated enhanced PTX permeation and greater cytotoxicity against U87-MG cells than free PTX and TMZ, although in vivo validation remains necessary. Similarly, intranasal RGD-conjugated, DOX-loaded PLGA nanoparticles produced tumor-selective accumulation and approximately 76% tumor-growth inhibition in a rat C6 glioma model while increasing apoptosis in the tumor region without detectable damage to normal brain tissue. Importantly, these PTX and DOX studies demonstrate the broader feasibility of intranasal chemotherapy, while the specific incorporation of chitosan remains an area requiring additional in vivo and translational validation [51,52,53].

### 6.2. Gene Therapy and RNA Interference RNA

Chitosan-based nanoparticles are particularly promising for intranasal delivery of nucleic-acid therapeutics because the positively charged polymer can electrostatically complex negatively charged RNA, protect nucleic acids against nuclease degradation, and facilitate cellular uptake. One of the most direct demonstrations was provided by Van Woensel et al., who developed chitosan nanoparticles loaded with siRNA against Galectin-1 (siGal-1), an immunosuppressive molecule overexpressed in GBM. Following intranasal administration, the nanoparticles reached intracranial tumors within hours and produced more than 50% reduction in Galectin-1 expression in tumor-bearing mice. In a subsequent study, the same group demonstrated that intranasal siGal-1/chitosan nanoparticles remodeled the GBM tumor microenvironment, reducing immunosuppressive myeloid and regulatory T-cell populations while increasing CD4^+^ and CD8^+^ T-cell responses; combination with TMZ or PD-1 blockade further increased survival in experimental GBM models [24].

CRISPR/Cas-based gene editing is a rapidly developing extension of this concept. Although effective CRISPR/Cas9 nanomedicines have been demonstrated for GBM using BBB-penetrating and tumor-targeted nanoparticles, direct evidence for intranasal chitosan-based CRISPR delivery to GBM remains limited. For example, BBB-penetrating CRISPR/Cas9 nanocapsules targeting PLK1 achieved up to 38.1% gene editing in brain tumors and extended median survival in mice, while another targeted CRISPR nanomedicine produced approximately 32% PLK1 gene knockout and increased median survival to 40 days. These studies provide a technological foundation for future intranasal chitosan–CRISPR systems, but such formulations should presently be regarded as a prospective research direction rather than an established intranasal therapeutic platform [54,55].

MicroRNA-based therapy represents another important application. Kumar et al. developed multifunctional gold–iron oxide nanoparticles coated with a β-cyclodextrin–chitosan hybrid polymer and loaded with miR-100 and anti-miR-21. Following intranasal administration in U87-MG orthotopic xenograft mice, the targeted nanoparticles accumulated in the tumor and, when combined with systemic TMZ, significantly prolonged survival compared with untreated controls, non-targeted nanoparticles, or TMZ alone [25]. Similarly, intranasally administered self-assembled nanoparticles containing antagomir-21 and a RAGE-antagonist peptide reduced miR-21 expression, increased pro-apoptotic proteins PTEN and PDCD4, reduced VEGF-associated angiogenesis, and produced antitumor effects in intracranial GBM models. These findings indicate that intranasal chitosan-containing systems can be used not only to transport cytotoxic drugs but also to modify oncogenic signaling and treatment resistance at the molecular level [52].

### 6.3. Immunotherapy and Phototherapy

The intranasal route may also be exploited to manipulate the immunological microenvironment of glioblastoma multiforme (GBM) while minimizing systemic exposure. A particularly relevant example is the intranasal delivery of chitosan nanoparticles carrying small interfering RNA (siRNA) against Galectin-1 (Gal-1). Rather than acting solely as a gene-silencing strategy, intranasal Gal-1 knockdown altered the immune composition of the tumor microenvironment, decreasing immunosuppressive myeloid cells and regulatory T cells while increasing CD4^+^ and CD8^+^ T-cell activity. Importantly, combining intranasal siGal-1 nanoparticles with dendritic-cell vaccination or programmed cell death protein 1 (PD-1) blockade generated synergistic antitumor responses in mouse models [52]. These findings suggest that chitosan-based nose-to-brain systems could potentially function as immune-modulating platforms that sensitize GBM to checkpoint inhibition rather than simply serving as passive drug carriers [52].

### 6.4. Comparative Analysis of Advanced Chitosan Strategies and Therapeutic Efficacy

The comparative evidence indicates that chitosan-based intranasal systems offer distinct formulation advantages, including enhanced nasal residence through mucoadhesive interactions, improved mucosal permeation, protection of labile molecular payloads, and the potential to combine chemotherapy with targeted molecular or immunological interventions. Experimental evidence is particularly well developed for small-molecule therapeutics like temozolomide (TMZ), carmustine, and gemcitabine, as well as advanced RNA-based gene therapies, whereas intranasal paclitaxel and doxorubicin studies have primarily employed RGD-functionalized PLGA nanoparticles rather than chitosan-based (Table 1) carriers [50,51].

A critical assessment of current literature reveals that the level of therapeutic efficacy achieved is directly dictated by the specific structural engineering of the chitosan matrix. Unmodified native chitosan platforms consistently exhibit the lowest direct nose-to-brain delivery efficiency due to their rapid clearance by mucociliary action within 15–20 min and their poor solubility at physiological pH levels above 6.5 [59,60,61,62,63]. Among single-derivative modifications, thiolated chitosan and quaternized trimethyl chitosan (TMC) achieve the greatest comparative efficacy, though through entirely different mechanisms. Thiolated chitosan excel in structural retention, forming covalent disulfide cross-links with mucosal glycoproteins that extend nasal residence times to over 4 to 6 h, driving a 3- to 5-fold increase in absolute brain bioavailability compared to native polymer matrices. In contrast, TMC platforms maximize transport kinetics; their permanent, pH-independent cationic charge actively maintains continuous electrostatic interactions with the mucosal lining while driving the transient opening of epithelial tight junctions to achieve the highest paracellular permeation rates along the olfactory and trigeminal nerve pathways [64,65].

The highest absolute therapeutic outcomes, measured by maximum tumor regression and significant prolongations in median post-treatment survival—are consistently produced by multi-functionalized hybrid configurations. Superimposing biological cell membranes or tumor-homing ligands onto a modified chitosan core (e.g., cell-membrane-wrapped TMC cores or transferrin-functionalized thiolated chitosan) successfully resolves the contradictory demands of the nose-to-brain axis. The core matrix overcomes initial mucosal clearance and barriers, while the outer biomimetic shell actively bypasses the blood–brain–tumor barrier (BBTB) to home directly into the glioblastoma microenvironment. In orthotopic glioblastoma models, these advanced hybrid carriers achieve up to an 8-fold increase in absolute targeting efficiency and extend post-treatment median survival times by more than 40% compared to standard systemic chemotherapeutic controls [66,67,68]. Nevertheless, most available studies remain strictly preclinical, and direct comparisons are heavily complicated by differences in nanoparticle composition, particle size, drug loading, dose, administration volume, animal species, tumor model, treatment duration, and efficacy endpoints. Future studies should therefore prioritize standardized pharmacokinetic and pharmacodynamic assessment, long-term nasal and neurological toxicity, dose reproducibility, device compatibility, and validation in clinically relevant models before these advanced nose-to-brain systems can transition into human clinical trials [63].

## 7. Translational Horizons: Challenges and Future Perspectives

### 7.1. Local Cytotoxicity and Physicochemical Correlates

The major translational concern associated with intranasal administration of chemotherapeutic nanocarriers is the possibility of local nasal toxicity. Although chitosan and several other nanocarrier components are generally considered biocompatible, their effects on the nasal mucosa depend strongly on formulation characteristics, particle size, surface properties, concentration, and exposure conditions. Critical evaluation of toxicological data indicates that specific structural parameters—namely, surface charge, degree of deacetylation (DD), and particle size—directly dictate cytotoxicity toward the nasal respiratory and olfactory epithelium [65].

The surface charge density, reflected by zeta potential, is a principal driver of structural cell membrane disruption. While a positive surface charge is essential to promote electrostatic mucoadhesion and transiently modulate epithelial tight junctions (via claudin-1 and ZO-1 redistribution), an excessively high cationic charge density, typically exceeding a zeta potential threshold of +30 mV, induces acute, concentration-dependent membrane perturbation, cellular enzyme leakage, and localized epithelial desquamation. This mechanism is deeply coupled with the polymer’s degree of deacetylation (DD) [66]. Chitosan formulations utilizing a highly deacetylated backbone (DD > 85%) present a dense concentration of protonated primary amino groups (NH3+). While this structural configuration optimizes drug transport kinetics, its chronic application accelerates cellular apoptotic pathways and compromises the mechanical integrity of the epithelial lining. Furthermore, nanoparticle geometry significantly influences tissue irritation; particles scaled below a critical diameter threshold of 50 nm exhibit elevated intracellular accumulation rates that can trigger intracellular reactive oxygen species (ROS) generation and localized inflammatory stress within ciliated epithelial cells [64,67].

Prolonged exposure to cytotoxic payloads may additionally produce epithelial irritation, altered ciliary function, inflammation, or injury to the olfactory region. The importance of evaluating ciliary function and epithelial integrity is supported by intranasal toxicity studies in which nasal epithelial morphology and ciliary-beat frequency (CBF) were used as vital indicators of local tolerability. Standardized ex vivo safety protocols dictate that an irreversible reduction in CBF exceeding 20% constitutes a definitive formulation toxicity failure [68]. Notably, chitosan hydrochloride and trimethyl chitosan did not produce major changes in ciliary-beat frequency under the tested conditions, whereas other absorption enhancers produced marked epithelial or ciliary damage. The potential for local toxicity is particularly important for intranasal delivery of anticancer agents because prolonged contact with formulations containing cytotoxic materials may cause ciliotoxicity, tissue damage, and irritation, emphasizing the need for systematic safety assessment of nose-to-brain chemotherapeutic formulations [34].

This issue is particularly important for repeated administration because preclinical studies frequently evaluate relatively short treatment periods that may not capture cumulative mucosal or olfactory toxicity. Consequently, future studies must incorporate repeated-dose histopathology, inflammatory biomarkers, ciliary-beat assessment, epithelial-barrier integrity, olfactory behavioural testing, and longer follow-up periods [69]. Crucially, chronic in vivo safety evaluations must transition to large animal models (such as rabbits or minipigs) whose nasal cavity volumes approximate human physiology, utilizing extended exposure timelines (28 to 90 days) to screen for mucosal atrophy or submucosal necrosis. The safety profile should be assessed for the complete formulation rather than chitosan alone because drug concentration, particle surface characteristics, preservatives, pH, osmolarity, particle size, and residence time may all influence nasal tolerability [34,35].

### 7.2. Volumetric and Dosing Constraints

A second important barrier is the limited fluid-handling capacity of the human nasal cavity. Conventional metered-dose nasal pumps commonly deliver approximately 25–200 μL per actuation, with approximately 100 μL being a typical delivered volume. Excessive administration volumes can promote rapid runoff toward the nasopharynx and swallowing, thereby reducing deposition at the olfactory region and increasing variability in the delivered dose. This constraint is particularly relevant for nanoparticles because effective treatment may require sufficiently high payload concentrations while maintaining an acceptable viscosity, osmolarity, and spreadability [70].

Chitosan-based hydrogels, concentrated nanoparticle suspensions, thermoresponsive systems, and in situ gelling formulations may help prolong residence time and reduce mucociliary loss; however, their rheological properties must remain compatible with reproducible device operation and efficient nasal administration. Therefore, future formulation development should focus not only on nanoparticle size and encapsulation efficiency but also on dose concentration, delivered volume, spray pattern, plume geometry, viscosity, droplet-size distribution, and regional deposition within the human nasal cavity [20].

### 7.3. Device and Device-to-Patient Translation

An important limitation in nose-to-brain research is the substantial difference between experimental administration in rodents and clinical nasal delivery in humans. In many animal studies, formulations are administered by pipetting small volumes directly into the nostrils, whereas clinical products require reproducible devices capable of generating a controlled plume and depositing a defined fraction of the dose in the desired nasal region [63]. Human translation will therefore require evaluation using metered-dose sprays, precision olfactory-targeting devices, or suitable nebulization systems. Device selection should be integrated into formulation development because spray force, droplet size, plume geometry, viscosity, and device characteristics can strongly influence nasal deposition and clearance [64].

Moreover, the human nasal cavity differs substantially from that of rodents in terms of anatomy, airflow patterns, olfactory-region distribution, mucociliary clearance, and nasal geometry [63]. These anatomical and physiological differences can limit the predictive value of conventional animal models for clinical nasal drug delivery. The complex and dynamic human nasal airway, including the narrow nasal valve and regional differences in airflow and deposition, represents an additional challenge for achieving reproducible delivery to the upper nasal and olfactory regions [6].

Consequently, successful translation will require comparative ex vivo human or porcine nasal models; computational fluid-dynamic (CFD) assessment of deposition, device–formulation compatibility studies; and, ultimately, clinical pharmacokinetic and imaging studies to demonstrate reproducible nasal deposition and brain targeting. CFD simulations and anatomically relevant nasal casts can provide useful information during device development, but their predictive value should ultimately be confirmed by human in vivo deposition and clinical studies. Integration of formulation properties with device design and clinically relevant administration conditions will therefore be essential for bridging the gap between promising preclinical nose-to-brain nanomedicine studies and reproducible patient-oriented delivery systems [3,8].

### 7.4. Regulatory Pathways and Scalable Manufacturing

The regulatory translation of chitosan-based nanomedicines will require robust control of formulation composition, manufacturing processes, critical quality attributes (CQAs), and product performance. Because these systems pair an active anticancer drug with a functional polymer that alters its pharmacokinetics and crosses neural barriers, regulatory bodies like the U.S. Food and Drug Administration (FDA) and the European Medicines Agency (EMA) classify them as drug-led combination products. For formulations utilizing previously approved chemotherapeutic agents encapsulated within novel chitosan matrices, the primary regulatory mechanism for clinical trial entry is the FDA’s 505(b)(2) New Drug Application (NDA) pathway, which significantly streamlines approval by leveraging existing safety data of the active innovator drug [68,69,70].

The FDA guidance on drug products containing nanomaterials emphasizes that nanomedicines require careful characterization of parameters such as particle size and size distribution, morphology, surface characteristics, composition, stability, and other properties that may influence safety and efficacy. Furthermore, the FDA’s broader guidance on nanotechnology-regulated products highlights the importance of evaluating nanoscale-related physicochemical properties and their potential implications for product safety, effectiveness, and regulatory classification [71,72].

## 8. Conclusions

Chitosan-based intranasal nanocarriers offer a promising approach to glioblastoma multiforme (GBM) treatment by improving drug delivery to the brain and providing a platform for delivering both conventional chemotherapeutic agents and newer molecular therapies. Despite encouraging preclinical findings, several challenges remain, particularly regarding safety, effective drug distribution, and treatment outcomes in humans. Further studies are needed to optimize formulations, establish reliable manufacturing processes, and evaluate their long-term safety and therapeutic effectiveness. Although these systems are not expected to replace current GBM treatments in the near future, they may provide a valuable complementary approach. With further research and successful clinical validation, intranasal chitosan-based nanocarriers could contribute to the development of more effective and targeted treatment strategies for patients with GBM.

## Figures and Tables

**Table 1:** Representative intranasal and chitosan-assisted nanocarrier systems for GBM.

Formulation	Payload	Model	Route	Main Outcome	Ref.
Nano-lipid chitosan hydrogel	Temozolomide (TMZ)	U87MG cells; nasal/animal evaluation	Intranasal	Particle size ~134 nm; IC_50_ 3.34 μg/mL versus 160 μg/mL for free TMZ; no structural damage to nasal mucosa was observed histopathologically	[48]
Chitosan-coated pH-sensitive liposomes	Temozolomide	U87MG cells; rats	Intranasal	Approximately 4.6-fold higher cellular uptake and >22-fold higher brain AUC than free TMZ; IC_50_ ~0.396 μg/mL	[56]
Chitosan-coated PLGA nanoparticles	Carmustine	U87MG cells; Albino Wistar rats	Intranasal	Brain AUC was 2.8-fold higher than intranasal carmustine suspension and 14.7-fold higher than intravenous administration	[23]
Chitosan-coated PLGA nanoparticles	Gemcitabine	U215 and T98G cells	Proposed/optimized for intranasal delivery	Enhanced mucoadhesion and antiproliferative activity; promoted sensitization of GBM cells to TMZ	[57]
Chitosan-siRNA nanoparticles	siGal-1	Murine/human GBM models; tumor-bearing mice	Intranasal	Galectin-1 knockdown altered the tumor microenvironment, reduced immunosuppressive myeloid and regulatory T-cell populations, increased CD4^+^/CD8^+^ T-cell activity, and improved survival; synergistic effects were observed with TMZ and immunotherapy	[24]
Chitosan-coated gold–iron oxide nanoparticles	miR-100 + anti-miR-21	U87-MG orthotopic GBM mice	Intranasal	Enhanced brain/tumor accumulation and sensitization to systemic TMZ, with prolonged survival in the combined treatment group	[52]
Self-assembled nanoparticles	Antagomir-21 + RAGE-antagonist peptide	Intracranial GBM animal model	Intranasal	Reduced miR-21 and RAGE signaling, increased PTEN/PDCD4 expression, enhanced apoptosis, and reduced angiogenic markers and tumor progression	[53]
RGD-PLGA nanoparticles	Paclitaxel	C6 GBM rat model	Intranasal	Approximately 75 ± 12% reduction in tumor burden, associated with enhanced apoptosis and/or inhibition of tumor-cell proliferation	[51]
RGD-PLGA nanoparticles	Doxorubicin	C6 GBM rat model	Intranasal	Approximately 76 ± 3.91% tumor-growth inhibition, reduced tumor burden, and enhanced apoptosis in the tumor region without evident damage to normal brain cells	[50]
Chitosan-coated lipid nanoparticles	AlClPc	U-87MG cells; mice	Nanocarrier/PDT	Enhanced reactive oxygen species generation, reduced GBM-cell viability and proliferation, and decreased total tumor area following photodynamic treatment without evident systemic toxicity	[58]

## Data Availability

The data supporting the findings of this study are available within the article.
